# Switching Correlation and Noise Level in Pr^3+^:YSO Crystal via Dressing Nonlinear Phase

**DOI:** 10.1038/srep33568

**Published:** 2016-09-21

**Authors:** Irfan Ahmed, Zhaoyang Zhang, Feng Wen, Da Zhang, Changbiao Li, Ruimin Wang, Yanpeng Zhang

**Affiliations:** 1 Key Laboratory for Physical Electronics and Devices of the Ministry of Education & School of Science & Shaanxi Key Lab of Information Photonic Technique, Xi’an Jiaotong University, Xi’an 710049, China; 2 Department of Electrical Engineering, Sukkur IBA, Sukkur 65200, Pakistan

## Abstract

We propose and experimentally demonstrate that the intensity noise correlation and the noise level of intensity-difference and intensity-sum in Stokes and anti-Stokes channel can be well controlled by the relative nonlinear phase in spontaneous parametric four-wave mixing process. By modulating the relative nonlinear phase, including self-phase modulation and cross-phase modulation, switching the correlation into anti-correlation and the relative intensity noise level between the intensity-difference and intensity-sum are realized. We also show that the variation tendencies of the relative intensity noise level and the corresponding intensity fluctuations correlation are in accordance with each other.

The experimental preparation, manipulation, and detection of correlated and entangled photon pairs are of great interest for research in fundamental physics and application of quantum information processing^1^,^2^,^3^. Conventionally, entangled photon pairs are produced from spontaneous parametric down-conversion (SPDC) in a nonlinear crystal, where a pump photon is annihilated and two down-converted daughter photons are simultaneously emitted^4^. Entangled photon pairs from spontaneous parametric four-wave mixing in atomic ensemble have also been generated^5^. Compared with the SPDC, the correlated biphotons produced from the SP-FWM have long coherence time (0.1–1.0 s) and narrow spectral width (~MHz)^6^,^7^,^8^. However, difficulty to integration and miniaturization limits the stepping toward integrated quantum photonic devices via SP-FWM in atomic ensemble. The rare-earth doped crystals, i.e. Pr^3+^:Y_2_SiO_3_, the “atom-like” properties of the dopant can be kept and the atomic coherence can be induced easily when interacting with multiple laser beams, which is different from traditional nonlinear crystal. Recent research progresses related to atomic coherence in such solid-state materials, including electromagnetically induced transparency (EIT)^9^, light velocity reduction and coherent storage^10^, all-optical routing based on optical storage^11^. Besides these, via nonlinear Kerr effects induced by atomic coherence, the correlation switching has been experimentally demonstrated^12^. Such modulation effect is governed by cross-phase modulated (XPM) and self-phase modulated (SPM) in Kerr nonlinear medium^13^.

In this paper, the intensity noise correlation and relative noise level of intensity-difference and intensity-sum in Stokes and anti-Stokes signals are investigated in Pr^3+^:Y_2_SiO_3_ crystal. We demonstrated that the switching the correlation into anti-correlation as well as the relative noise about the intensity-difference noise level (IDNL) and intensity-sum noise level (ISNL) can be realized by modulating the relative nonlinear phase, including self-phase modulation and cross-phase modulation. Especially, the correlation and relative noise level can be manipulated by power and polarization of the dressing field participating in the SP-FWM process.

## Results

**Figure 1:**
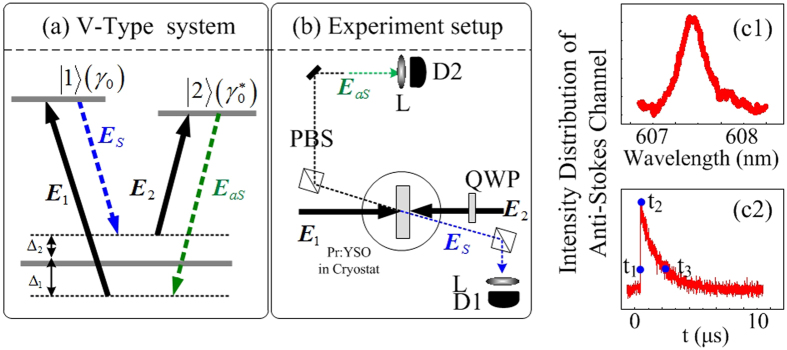
(**a**) Three-level system (V-type) in Pr^3+^:YSO crystal and the laser coupling configuration. (**b**) Experimental setup scheme. D: photomultiplier tube, PBS: polarized beam splitter, BS: beam splitter, and L: lens, QWP: quarter wave plate. (**c1,c2**) shows intensity distribution of anti-Stokes in frequency and time domain, respectively.

Figure 1(a) gives the schematic diagram of the experimental arrangement, where the sample (a 3-mm Pr^3+^:Y_2_SiO_3_ crystal) is held at 77 K in a cryostat (CFM-102). two tunable dye lasers (with a 0.04 cm^−1^ linewidth) pumped by an injection locked single-mode Nd:YAG laser (Continuum Powerlite DLS 9010, 10 Hz repetition rate, 5 ns pulse width) are used to generate the pumping fields *E*_1_ (*ω*_1_, Δ_1_) and *E*_2_ (*ω*_2_, Δ_2_) with the frequency detuning of Δ_*i*_ = *ω*_*mn*_ − *ω*_*i*_ (i = 1, 2), respectively, where Ω_*mn*_ is the transition frequency between level 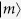 and 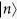. *E*_1_ drives the transition 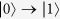, and *E*_2_ couples to the transition 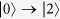. Specifically, the horizontally polarized pumping beam *E*_1_ with peak power of about 6 kW and beam waist of 280 μm is combined with pumping beam *E*_2_ (about 12 kW) at an angle of 3 mrad at the center of Pr^3+^:Y_2_SiO_3_ crystal, where *E*_1_ crosses with *E*_2_ and amplified with a gain of G≈3.5. Based on these settings, two narrow band correlated photons pairs (Stokes *E*_*S*_ and anti-Stokes *E*_*AS*_) are generated via SP-FWM process, and the corresponding photon counting rate for Stokes and anti-Stokes channel are 1 × 10^6^ *s*^−1^ and 6 × 10^7^ *s*^−1^, respectively.

When two pumping field *E*_1_ and *E*_2_ are switched on, in the V-type three-level 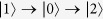, two narrow band correlated photons (Stokes field *E*_*S*_ and anti-Stokes field *E*_*AS*_) are generated via SP-FWM process, satisfying the phase-matching condition ***k***_1_ + ***k***_2_ = ***k***_*AS*_ + ***k***_*S*_. Figure 1(b) shows the experimental arrangement taking into account the above phase-matching conditions. The generated Stokes signal *E*_*S*_ and anti-Stokes signal *E*_*AS*_ are reflected by polarized beam splitters (PBS) and detected by photomultiplier tubes, and recorded by D_1_ and D_2_. To study fluctuations in stokes and anti-stokes channel transmitted through Pr^3+^:YSO, the dependence of the intensities on time and frequency are registered by digital oscilloscope and photomultiplier tubes, respectively, as shown in Fig. 1(c1, and then the obtained noises in stokes and anti-stokes channel are analyzed by two methods. On one hand, by using intensity fluctuations *δI*_*i*_(t) (*i* = *AS*, *S*) recorded by digital oscilloscope, the temporal waveform correlations between stokes and anti-stokes channel are investigated by Eq. (6) under a variety of conditions. On the other hand, by using intensity fluctuations recorded by photomultiplier tubes, the measured intensity fluctuations in stokes and anti-stokes channel are subtracted and added from each other, and are then analyzed with a spectrum analyzer to investigate the relative noise power between intensity-difference and intensity-sum, as indicated by Eq. (7). Here, Fig. 1(c1 show the measured intensity of anti-Stokes signal in frequency and time domain, respectively.

In the interaction picture, the coupled Stokes channel and anti-Stokes channel can be described by


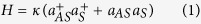


where 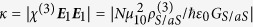 and *κ* is called the coupling parameter of the SP-FWM, depending on the nonlinearity coefficient *χ*^(3)^ and intensity of the pump field, and 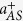 (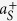) is boson annihilation operator acting on *E*_*AS*_ (*E*_*S*_) channel, *G*_*Si*_ = *μ*_*k*_***E***_*Si*_*/ħ Rabi frequency of **E***_*Si*_.(*i* = *1, 2, 3*)*, μ*_*ij*_ *electric dipole moment between energy state* 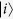 *and* 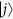
*, and N averaged atomic density*. According to the Liouville pathways [8], via the dressed perturbation chains of 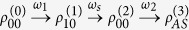 (*E*_*AS*_) and 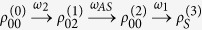 (*E*_*S*_), the density-matrix element for *E*_*S*_ and *E*_*AS*_ can be expressed as 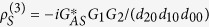 and 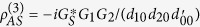, where *G*_*i*_ = *μ*_*i*_*E*_*i*_/*ħ* (i = 1, 2) is the Rabi frequency of the field *E*_*i*_, and Γ_*ij*_ is the decay rate between the energy levels 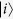 and 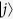. *d*_20_ = Γ_20_ + *i*Δ_2_, *d*_10_ = Γ_10_ + *i*Δ_1_, *d*_00_ = Γ_00_ + *i*(Δ_2_ − Δ_1_), 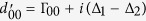.

Next, taking the dressing effects of *E*_1_ and *E*_2_ into account, the revised density-matrix element for *E*_*S*_ and *E*_*AS*_ can be rewritten as


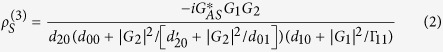



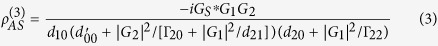


where 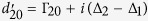, *d*_21_ = Γ_21_ − *i*Δ_1_, *d*_01_ = Γ_01_ − *i*Δ_1_.

In addition, the polarization of *E*_2_ is modulated by inserting a quarter-wave plate (QW) in *E*_2_ beam (see Fig. 1(b)), while pumping field ***E***_1_ is kept linearly polarized. The vertically polarized components (S-polarization) of Stokes and anti-Stokes signals reflected by PBS are detected. Therefore, the effective density matrix elements for *E*_*S*_ and *E*_*AS*_ are given by


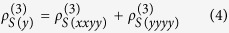



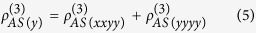


In this case, the Rabi frequency |*G*_2_|^2^ in Eq. (2) is replaced by 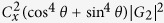 for matrix element 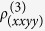 and 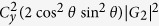 for 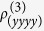, respectively. Where *c*_*x*,*y*_ is the anisotropic factor in different directions of crystal, *θ* is the rotated angle of the QWP’s axis from the *x* axis.

Based on the measurements of the second-order coherence functions, See Methods for theoretical derivations of the intensity distribution at each output channel, the intensity fluctuations correlation between the Stokes and anti-Stokes signals^14^,^15^,^16^ can be obtained as


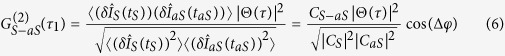


where averaging over the time is defined as 
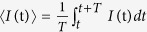
, τ = t_*S*_ − t_*AS*_ is the selected time delay between Stokes and anti-Stokes signal, and *T* is the time of integration, in our case *T* = 10 *μ*s. Δ*φ* = *φ*_*S*_ − *φ*_*AS*_ Is relative nonlinear phase between Stokes and anti-Stokes signal based on XPM and SPM.

Different from the intensity-difference squeezing^15^, we investigate the relative noise power between the intensity-difference and intensity-sum in V-type systems, which can be modified as^17^,^18^,^19^


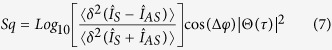


where 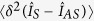 and 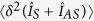 are mean square deviation of intensity fluctuations difference and sums between Stokes and anti-Stokes signal, respectively, Θ(*τ*) is two photon envelop function.

To study the role of the relative nonlinear phase played on intensity fluctuations correlation as well as the intensity noise level between the IDNL and ISNL of anti-Stokes and Stokes beams, the dependence of the intensities on time 〈*I*_*i*_〉 + *δI*_*i*_(t) (*i* = *AS*, *S*) are registered. Here 〈*I*_*i*_〉 is the average intensity of anti-Stokes/Stokes beams, and *δI*_*i*_(t) are corresponding intensity fluctuations. After the intensity fluctuations is averaged by a fast gated integrator over 10 pulses, and then using the averaged data, the correlation curve is calculated via cross correlation of the intensity fluctuation in stokes and anti-stokes channel.

**Figure 2:**
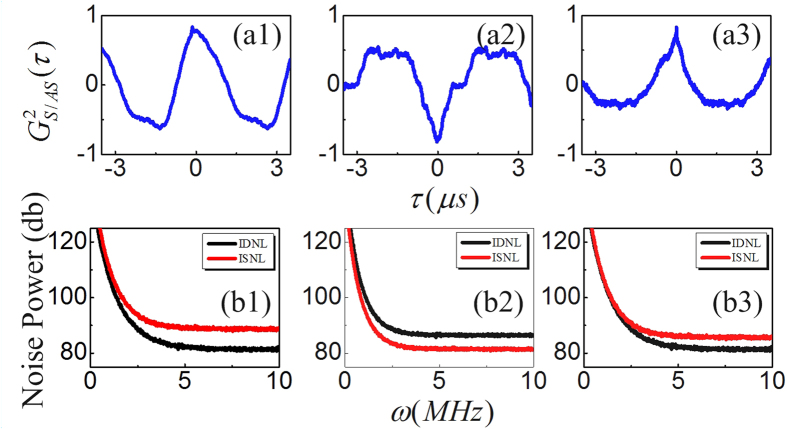
The noise correlations and the relative intensity noise level between IDNL and ISNL of *E*_*AS*_ and *E*_*S*_ are shown in (**a1–a3,b1–b3**), respectively, with *P*_1_ = 4 mW, and *P*_2_ = 6, 8, and 10 mW.

Figure 2(a) shows the fluctuation correlations between *E*_*AS*_ and *E*_*S*_ under different dressing powers, where the curve is calculated by substituting the recorded intensity fluctuations *δI*_*AS*,*S*_(t) into Eq. (6) setting Δ_1_ = Δ_2_ = 0 and *P*_1_ = 4 mW. When *P*_2_ = 6 mW, as shown in Fig. 2(a1), the correlation peak 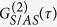 at delay time *τ* = 0 has amplitude of 0.83 ± 0.01 and corresponding full width at half maximum (FWHM) is 2.2 *μs*. If we set the power of *E*_2_ at *P*_2_ = 8 mW, as shown in Fig. 2(a2), we find the amplitude of correlation peak 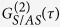 at *τ* = 0 is switched from positive (0.83 ± 0.01) to negative (−0.82 ± 0.01), therefore, the intensity fluctuations is changed from correlated to anti-correlated. The FWHM of the correlation peaks has increased to 1.8 *μs*. In contrast, at *P*_2_ = 10 mW, the correlation peak 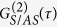 at *τ* = 0 is adjusted back to correlated with amplitude 0.83 ± 0.01 and corresponding FWHM is further increased to 1.5 *μs*, as shown in Fig. 2(a3). These results may be explained by nonlinear refractive index of Kerr medium. Since the dressing state (created by *E*_2_) can modulate the nonlinear refractive index of Kerr medium and lead to XPM, the relative nonlinear phase between Stokes and anti-Stokes signal 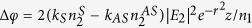 can be significantly modulated. Where Δ*φ* = *φ*_*S*_ − *φ*_*AS*_ is the relative nonlinear phase between Stokes and anti-Stokes signal based on XPM, whereas *φ*_*S*_ (*φ*_*AS*_) is nonlinear phases induced on Stokes (anti-Stokes) signal, respectively and *r* is the beam radium of Gaussian beam incident from laser and *z* is length of YSO crystal’s lens (z = 3 mm). Specifically, by changing the power of *E*_2_ at 6, 8, and 10 mW, the relative nonlinear phase between Stokes and anti-Stokes are modulated to Δ*φ* = 0, *π*, and 2*π*, respectively. Therefore, 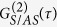 at *P*_2_ = 6 mW and 10 mW have positive correlation peak, and at *P*_2_ = 8 mW have negative correlation peak.

On the other hand, by substituting *δI*_*AS*,*S*_(t) into Eq. (7), the influence of the nonlinear phase on the relative intensity noise level between the intensity-difference and intensity-sum are also investigated. To measure the relative noise level between the IDNL and ISNL, we use two balance detector to pick up the noise in stokes and anti-stokes channel, where the two coherent beams (not shown in Fig. 1), one have the same intensity with stokes and another have same intensity with anti-stokes, are used to illuminate to two detectors during measurement. The measured noise in stokes and anti-stokes are subtracted and added from each other, and then analyzed with a spectrum analyzer. Figure 2(b1–b3) demonstrated that noise level of the intensity-difference signal (IDNL, black curve) can be switched higher or lower than the intensity-sum signal (ISNL, red curve) by changing Δ*φ* = 0, *π*, and 2*π*, respectively. we can see that the noise level of intensity-difference signal is lower than the intensity-sum signal when Δ*φ* = 0 and 2*π*, as in Fig. 2(b1, while the relative noise level of intensity-difference is switched higher than intensity-sum when Δ*φ* = *π*, see Fig. 2(b2). It is worth mentioning that the variation tendencies of the intensity noise level between the intensity-difference and intensity-sum and the corresponding intensity fluctuations correlation are in accordance with each other. Therefore, intensity fluctuations correlation and relative intensity noise level depends crucially on the relative nonlinear phase induced by XPM.

The modulation of the nonlinear phase can be realized not only by changing the power of *E*_2_, but changing the polarization of *E*_2_ as well. As stated above, the polarization of the dressing field *E*_2_ can be controlled by inserting a QWP, and the effective Rabi frequency of *E*_2_ for matrix element 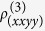 is 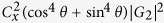, and for 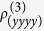 is 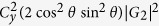. Based on above discussion, the matrix element 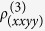 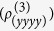 and corresponding third-order effective susceptibility can be modulated by the rotating the angle of QWP therefore, the nonlinear phase to detected signal can also be modulated.

**Figure 3:**
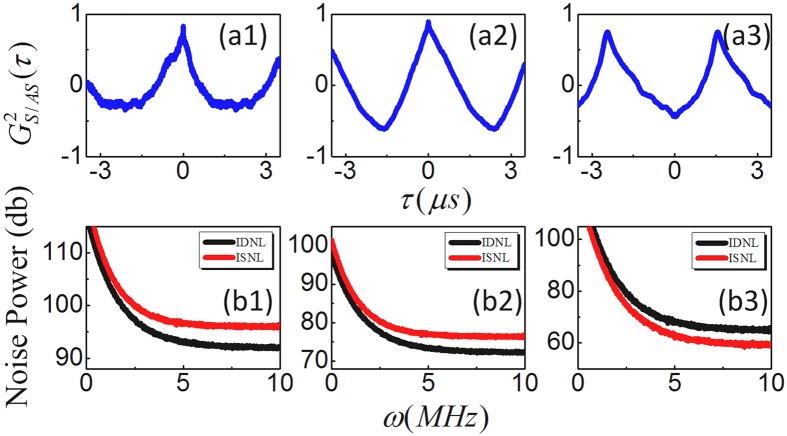
Same as Fig. 2 but with changing polarization of E_2_ from linear (**a1,b1**), elliptical (**a2,b2**), and to circular (**a3,b3**).

Now, we focus on the polarization dependences of the intensity fluctuations correlation and the relative intensity noise level between IDNL and ISNL. Modulated polarization can be achieved by inserting QWP in Fig. 1(b) in front of incident beam *E*_2_. Figure 3(a1–a3) show the correlation curves at linear polarization (*θ* = 0), elliptical polarization (*θ* = *π*/6), and circular polarization (*θ* = *π*/4), respectively, with setting Δ_1_ = Δ_2_ = 0, *P*_1_ = 5 mW and *P*_2_ = 10 mW. We can see from Fig. 3(a1–a3) that intensity fluctuations is changed from correlated to anti-correlated as polarization modulated from linear to circular polarization. In addition, the relative intensity noise level between the IDNL and ISNL of the output beams *E*_*AS*_ and *E*_*S*_ depends crucially on the correlation functions, therefore, the corresponding relative noise level of intensity-difference is switched from lower than the intensity-sum to higher than the intensity-sum as polarization modulated from linear to circular. This phenomenon can be interpreted from Eqs (2)–(5), 20, 22, 23, where relative nonlinear phase 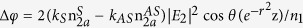 (*a* = 0, +, and − are corresponding to linear, left circular, and right circular polarization, respectively.) induced by XPM at circular polarization is about *π*, and at linear (*θ* = 0) and elliptical (*θ* = *π*/6) polarization is about 0 and 2*π*. Therefore, relative intensity noise level between IDNL and ISNL and correlation can also be switched by polarization of pumping field.

## Discussion

**Figure 4:**
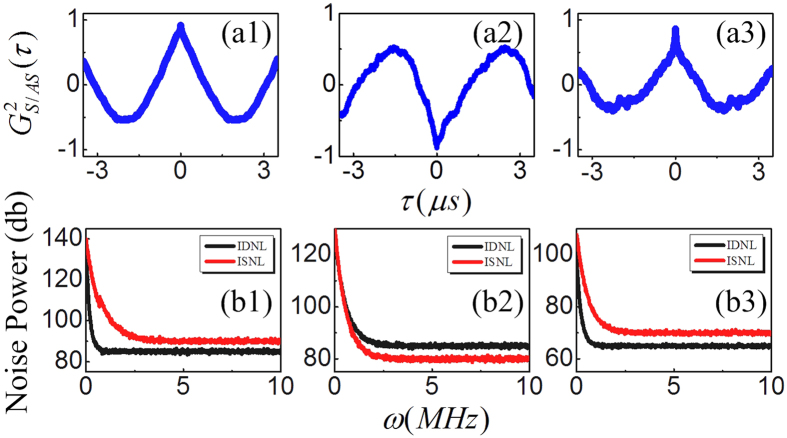
By selecting *t*_1_, *t*_2_, and *t*_3_ in decay curves as shown in Fig. 1(c2), the noise correlations and the relative intensity noise between IDNL and ISNL are demonstrated in (**a1–a3,b1–b3**), respectively.

Finally, by selecting different timing position in decay curves, see Fig. 1(c2), the noise correlations and relative intensity noise level between the IDNL and ISNL of the output beams *E*_*AS*_ and *E*_*S*_ are demonstrated, as shown in Fig. 4(a,b), respectively, where those curves are calculated under fixing Δ_1_ = Δ_2_ = 0 and setting *P*_1_ = 5 mW and *P*_2_ = 10 mW. At the high intensity time position (2^nd^), 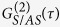 is obtained as shown in Fig. 4(a2), where the correlation peak at *τ* = 0 has amplitude −0.88 ± 0.01 and corresponding FWHM is 1.6 *μs*. It is interesting that the correlation is switched to correlation with amplitude 0.86 ± 0.01 and 0.89 ± 0.01 at the lower intensity time position (1^st^) and (3^rd^), respectively. This phenomenon are tightly related to the intensity of generated field *E*_*AS*_ and *E*_*S*_, and can be explained by relative nonlinear phase induced by SPM 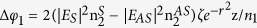, here *ζ* = |*G*_2_/*G*_*S*/*AS*_|^2^ is intensity ratio of strong field *G*_2_ and obtained weak fields 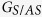. The SPM is significantly at 2^nd^ for intensity of *E*_*AS*_ and *E*_*S*_ are the largest at this timing point, therefore, the relative phase induced by SPM at 2^nd^ is about Δ*φ* ≅ *π*. On the other hand, at 1^st^ and 3^rd^, the SPM can be ignored for faint intensity of *E*_*AS*_ and *E*_*S*_, therefore, the relative phase between Stokes and anti-Stokes is same Δ*φ* ≅ 0, which is the reason that Fig. 4(a1 are almost symmetrical to each other. With the same reason, the IDNL is demonstrating significantly higher than intensity-sum signal at 2^nd^ (Fig. 4(b3)), and lower than ISNL at 1^st^ and 3^rd^. Therefore, intensity fluctuations correlation and the relative intensity noise level between IDNL and ISNL also depend crucially on the relative nonlinear phase induced by SPM.

The dressed noise correlation and the relative intensity noise level between IDNL and ISNL based on SP-FWM process in Pr^3+^:Y_2_SiO_3_ crystals have been observed experimentally and explained theoretically. We observed that the correlation and the relative intensity noise level between IDNL and ISNL can be switched by controlling the power and polarization of the dressing field. These results are attributed to dressing-induced self-phase modulation and cross-phase modulation. The investigation may find applications in all-solids quantum communication and quantum information processing.

## Methods

Experimental setup. the sample used in present experiments is a 0.05% rare-earth Pr^3+^ doped Y_2_SiO_3_ (Pr^3+^:Y_2_SiO_3_) crystal, and the triplet energy-level (^3^*H*_4_) and singlet energy-level (^1^*D*_2_) of Pr^3+^ are selected which can be distinguished by investigating the optical spectrum of Pr^3+^. The degeneracy of the energy levels of the Pr^3+^ ion is removed by the crystal field of YSO, where the terms in ^3^*H*_4_ and ^1^*D*_2_ states are split into nine and five Stark components, respectively. The Pr^3+^ impurity ions occupy two nonequivalent cation sites (sites I and II, respectively) in the YSO crystal lattice. The energy levels are labeled by a Greek letters with and without asterisk for site II and I sites, respectively. The coupling between Pr^3+^ ions localized at different cation vacancies can happen due to induced dipole-dipole interactions, so one can treat the two ions as a hetero-nuclear-like molecule. Therefore, we can construct a V-type three-level subsystem (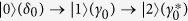) by coupling the corresponding laser fields as shown in Fig. 1(a).

### Theoretical models for the intensity distribution in each output channel

When the generated Stokes and anti-Stokes signals passes through the Pr^3+^:Y_2_SiO_3_ crystal along the z axis, the nonlinear cross-phase modulation as well as self-phase modulation is acquired. According to Eq. (1), the propagation dynamics for 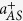 and 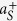 at the output surface of is determined by^20^−^22^,


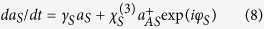



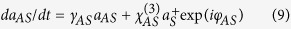


where nonlinear susceptibility coefficients 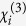 is proportional to 
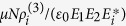
. *φ*_*S*_ (*φ*_*AS*_) is nonlinear phases induced on Stokes (anti-Stokes) signal, respectively, and the relative nonlinear phase between Stokes and anti-Stokes signal can be defined as Δ*φ* = *φ*_*S*_ − *φ*_*AS*_. Here, the induced nonlinear phase shift Δ*φ* can be defined as 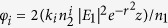, where 
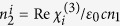
 is self or cross-Kerr nonlinear coefficient for Stokes (anti-Stokes) field, and *n*_1_ is the linear refractive index. Therefore, from Eqs (4) and (5), the photon number from output of Stokes/anti-Stokes channel can be solved as


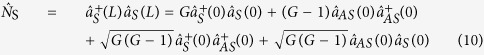



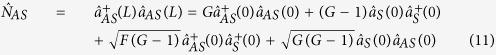


where the gain coefficient 
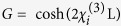
 is deduced as function of nonlinear susceptibility, and the intensity at each output channel proportional to the measured photon number can be written as 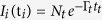.

According to the Liouville pathways 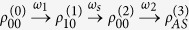 (*E*_*AS*_) and 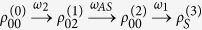 (*E*_*S*_), the non-steady state solutions of density-matrix element for *E*_*S*_ and *E*_*AS*_ can be expressed as


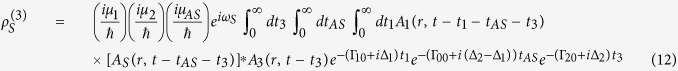



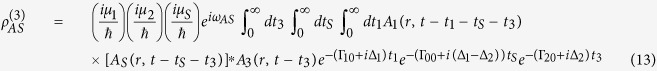


where 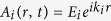.

In addition, taking the population distributions in levels |1〉 and |2〉 into account, where the Boltzmann distribution with finite temperature is considered in the deduction, the two-photon envelope function Θ(*τ*) is obtained as


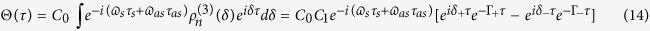


where *C*_0_ and *C*_1_ are time independent constants, and *n* = *AS*/*S* and 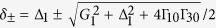. The first term in the square brackets on the right-hand side of Eq. (14) represents the two-photon amplitude between correlated photons peaking at *ϖ*_*s*_ = *ω*_1_ − *δ*_+_ and *ϖ*_*AS*_ = *ω*_1_ + *δ*_+_ with linewidth Γ_+_, while the second term stands for the two-photon amplitude between paired photons centered at *ϖ*_*s*_ = *ω*_1_ − *δ*_+_ and *ϖ*_*AS*_ = *ω*_1_ + *δ*_+_ with linewidth Γ_−_. The sum of these two amplitudes is manifested by a slowly oscillating phase term.

**Figure 5:**
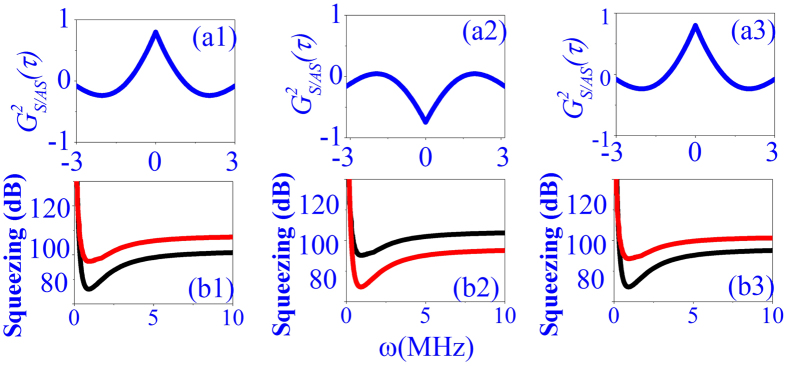
The simulated plots of noise correlations and the relative intensity noise level between IDNL and ISNL of *E*_*AS*_ and *E*_*S*_ are shown in (**a1–a3,b1–b3**), respectively in accordance with experimental conditions defined in Fig. 2.

Finally, the simulated plots of noise correlations and the relative intensity noise level between IDNL and ISNL of *E*_*AS*_ and *E*_*S*_ are shown in Fig. 5(a1–a3, respectively in accordance with experimental conditions defined in Fig. 2 and have been calculated using Eq. (6) involving relative non linear phase. Figure 5(a1–a3), shows simulation plots of second order correlation function 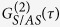 at delay time *τ* = 0. The results of correlation in Fig. 2(a1–a3) precisely match the simulated plots involving the phase shift in Fig. 5(a1–a3). Besides, Fig. 5(a1–a3) shown the theoretical plots for relative intensity noise level between IDNL and ISNL. The simulated plots of IDNL and ISNL precisely matches with obtained experimental plots of IDNL and ISNL and are calculated using Eq. (7).

## Additional Information

**How to cite this article**: Ahmed, I. *et al*. Switching Correlation and Noise Level in Pr^3+^:YSO Crystal via Dressing Nonlinear Phase. *Sci. Rep.* **6**, 33568; doi: 10.1038/srep33568 (2016).
